# Patient-, health worker-, and health facility-level determinants of correct malaria case management at publicly funded health facilities in Malawi: results from a nationally representative health facility survey

**DOI:** 10.1186/1475-2875-13-64

**Published:** 2014-02-20

**Authors:** Laura C Steinhardt, Jobiba Chinkhumba, Adam Wolkon, Madalitso Luka, Misheck Luhanga, John Sande, Jessica Oyugi, Doreen Ali, Don Mathanga, Jacek Skarbinski

**Affiliations:** 1 Malaria Branch, Division of Parasitic Diseases and Malaria, Centers for Disease Control and Prevention, Atlanta, GA, USA; 2 Malaria Alert Centre, College of Medicine, Blantyre, Malawi; 3 National Malaria Control Programme, Ministry of Health, Lilongwe, Malawi; 4 President’s Malaria Initiative, and Malaria Branch, Division of Parasitic Diseases and Malaria, Centers for Disease Control and Prevention, Lilongwe, Malawi

**Keywords:** Malaria, Case management, Symptoms, Diagnostics, Treatment, Malawi

## Abstract

**Background:**

Prompt and effective case management is needed to reduce malaria morbidity and mortality. However, malaria diagnosis and treatment is a multistep process that remains problematic in many settings, resulting in missed opportunities for effective treatment as well as overtreatment of patients without malaria.

**Methods:**

Prior to the widespread roll-out of malaria rapid diagnostic tests (RDTs) in late 2011, a national, cross-sectional, complex-sample, health facility survey was conducted in Malawi to assess patient-, health worker-, and health facility-level factors associated with malaria case management quality using multivariate Poisson regression models.

**Results:**

Among the 2,019 patients surveyed, 34% had confirmed malaria defined as presence of fever and parasitaemia on a reference blood smear. Sixty-seven per cent of patients with confirmed malaria were correctly prescribed the first-line anti-malarial, with most cases of incorrect treatment due to missed diagnosis; 31% of patients without confirmed malaria were overtreated with an anti-malarial. More than one-quarter of patients were not assessed for fever or history of fever by health workers. The most important determinants of correct malaria case management were patient-level clinical symptoms, such as spontaneous complaint of fever to health workers, which increased both correct treatment and overtreatment by 72 and 210%, respectively (p < 0.0001). Complaint of cough was associated with a 27% decreased likelihood of correct malaria treatment (p = 0.001). Lower-level cadres of health workers were more likely to prescribe anti-malarials for patients, increasing the likelihood of both correct treatment and overtreatment, but no other health worker or health facility-level factors were significantly associated with case management quality.

**Conclusions:**

Introduction of RDTs holds potential to improve malaria case management in Malawi, but health workers must systematically assess all patients for fever, and then test and treat accordingly, otherwise, malaria control programmes might miss an opportunity to dramatically improve malaria case management, despite better diagnostic tools.

## Background

Although much progress has been made in reducing malaria morbidity and mortality worldwide in the last decade, malaria still remains the third leading cause of death among children aged one to 59 months [1] and was responsible for an estimated 207 million cases worldwide in 2012 [2]. At the individual level, prompt and effective malaria case management can prevent progression to severe disease and death [3], and reduce the risk of anaemia from chronic infection. At the population level, appropriate case management might curtail malaria transmission by reducing the human parasite reservoir and prevent emergence of drug-resistant parasite strains [4-6]. Improving malaria case management may also contribute to improved treatment of non-malarial febrile illnesses, which are often misdiagnosed and treated presumptively as malaria [7].

Despite its importance, significant shortcomings persist in malaria case management [8], and a variety of factors contribute to these deficiencies [9]. Previous research showed that clinical algorithms for diagnosing malaria were largely inaccurate [10,11], but it was not until the widespread scale-up of rapid diagnostic tests (RDTs) for malaria after 2010 that researchers began to systematically investigate the myriad factors that influence case management quality.

In 2010, Malawi was one of 14 countries contributing to 80% of the estimated malaria cases worldwide [4] and reported about seven million suspected cases of malaria treated annually at public health facilities [12]. Despite Malawi’s high malaria burden, little is currently known about the quality of malaria case management in its publicly funded facilities, where the majority of Malawians seek care for fever [13]. Until late 2011, Malawi still recommended presumptive diagnosis and treatment for malaria of all febrile children and of adults when microscopy was not available. A nationally representative, complex sample survey was conducted during peak malaria transmission in Malawi in April-May 2011 among patients seeking care at outpatient departments of public facilities in Malawi, to evaluate the determinants of correct malaria diagnosis and treatment, including the relative contributions of patient-, clinician-, and health facility-level factors.

## Methods

### Study setting

Malaria is endemic and remains moderate to high transmission in each of Malawi’s three regions (Northern, Central, Southern), despite several years of scaling up malaria control interventions [14]. National surveys showed that malaria parasitaemia prevalence among children age six to 59 months was 43% in 2010 [15] and 28% in 2012 [16]. The majority of patients with fever seek treatment at publicly funded health facilities, including government facilities, which provide care free of charge, and at Christian Health Association of Malawi (CHAM) facilities, which are mission-run and charge a small fee to patients. At the first level, health centres provide primary care services, and community hospitals, also called rural hospitals, provide both primary and secondary care. At the second level, district hospitals serve as referral facilities for health centres and community hospitals and provide primary care to their own catchment areas [17]. The majority of primary care at health centres is provided by medical assistants, a position with two years of training. Clinical officers, a position with three years of training, provide some primary care at community and district hospitals [17].

Malawi has historically demonstrated leadership in malaria case management, becoming the first sub-Saharan African country in 1993 to change its first-line treatment from chloroquine to sulphadoxine-pyrimethamine (SP) [18]. In 2007, Malawi once again changed from SP to the artemisinin-based combination therapy (ACT) artemether-lumefantrine (AL). Second-line treatment for uncomplicated malaria, in case of AL failure, is artesunate-amodiaquine.

### Sampling and survey population

A three-stage, cluster sampling approach was used to select patients for the survey from: 1) a stratified random sample of all government of Malawi and CHAM health facilities (four facilities selected per district); 2) one outpatient department (OPD) per facility; and, 3) a systematic random sample of all patients presenting to the selected OPD on the day of the survey team’s visit during regular working hours (07:30–17:00). Additional details of the sampling are provided elsewhere [19]. All eligible patients who consented to participate before seeing the clinician were given a study card on which the health workers recorded a unique health worker identification number, the results of any laboratory tests ordered, and the diagnoses given to the patient. Patients were interviewed by the study team after they attended their consultation and collected any drugs prescribed. The survey team then administered a questionnaire about their symptoms and clinical encounter and performed a brief re-examination, including a thick and thin reference blood smear. Drugs prescribed were noted from information recorded in patient health passports, drug packs dispensed to patients, or prescriptions written by the health worker. Blood smears taken during the exit interview were double-read by expert microscopists at the Malaria Alert Centre in Blantyre, with a third reading for smears with discrepant first and second readings.

In addition, all health workers providing clinical consultations in the selected OPD on the day of the visit were interviewed about their training, access to malaria guidelines, and supervision. Finally, a health facility assessment at each sampled facility was conducted through interviews of the facility in-charge and direct observation and data collected on facility equipment, staffing and infrastructure.

### Outcome definition and statistical analysis

The primary outcome was correct treatment of confirmed uncomplicated malaria. Confirmed malaria was defined as parasitaemia on the reference blood smear, plus measured fever (temperature on re-examination ≥37.5°C) or history of fever, defined as one or more of the following: 1) patient reported during the exit interview that their illness involved a fever; 2) patient spontaneously mentioned fever complaint to health worker; or, 3) patient reported a symptom of fever to the surveyor when probed. Correct treatment for patients with confirmed malaria was defined as prescription of an appropriate anti-malarial. In most cases this was ACT (AL or artesunate-amodiaquine), but oral quinine for those weighing <5 kg or for pregnant women in their first trimester. A secondary outcome was overtreatment, defined as prescription of ACT (or other appropriate anti-malarial) to patients without confirmed malaria. Factors related to health worker diagnosis of malaria, an important mediator of correct malaria treatment in a setting where most patients are diagnosed presumptively, were analysed.

All patients were supposed to be assessed for fever. When another obvious cause of fever was not present, febrile patients (all children and adults when microscopy was not available) should have been prescribed an appropriate anti-malarial. Although some previous studies of malaria case management prior to diagnostics scale-up have assessed adherence to clinical guidelines, including presumptive treatment [20,21], these analyses used a more outcome-based approach of whether patients with confirmed malaria received correct treatment, and whether patients without confirmed malaria were prescribed an anti-malarial (overtreatment) [22]. While one would expect to see a fair amount of overtreatment using this approach in a setting without universal diagnostics, correct treatment of patients with confirmed malaria, arguably the more important outcome, should be very high if health workers were following the guidelines.

Frequencies and cross-tabulations were calculated using the survey commands in Stata Version 11.0 (College Station, TX, USA) to account for the complex survey design, including clustering at the health facility level. Sample weights were also used to generate indicators representative at national levels. Key case management variables were tested for differences by patient age (<five *versus* ≥ five years) using a Chi-square test with the Rao-Scott correction for survey data. Predictors of the outcomes of interest were examined at the patient, health worker, health facility, and regional levels using Poisson regression to calculate prevalence ratios, which some biostatisticians have posited are more appropriate than odds ratios for analysing non-rare events in cross-sectional surveys [23]. Predictors found to be significant in bivariate analyses at the p < 0.10 level were included in multivariate regressions. Plausible interaction terms were tested and kept in the model if significant. Potential confounders were then added to the model one at a time and kept in if they changed any of the other significant predictors by 20% or more.

### Ethical approval

Individual, written, informed consent was obtained from all eligible patients, and verbal consent from health workers before conducting interviews. Consent for children aged <18 years was obtained from the guardian or parent. For patients aged seven to 17 years, assent was also obtained from the patient in addition to consent from the guardian or parent. In an effort to maintain confidentiality, participants’ data were linked to a unique identifier, and patient names were not recorded. The Malawi College of Medicine Ethical Committee and the Centers for Disease Control and Prevention reviewed and approved the protocol prior to data collection.

## Results

### Patient characteristics and health worker assessment and treatment

**Figure 1:**
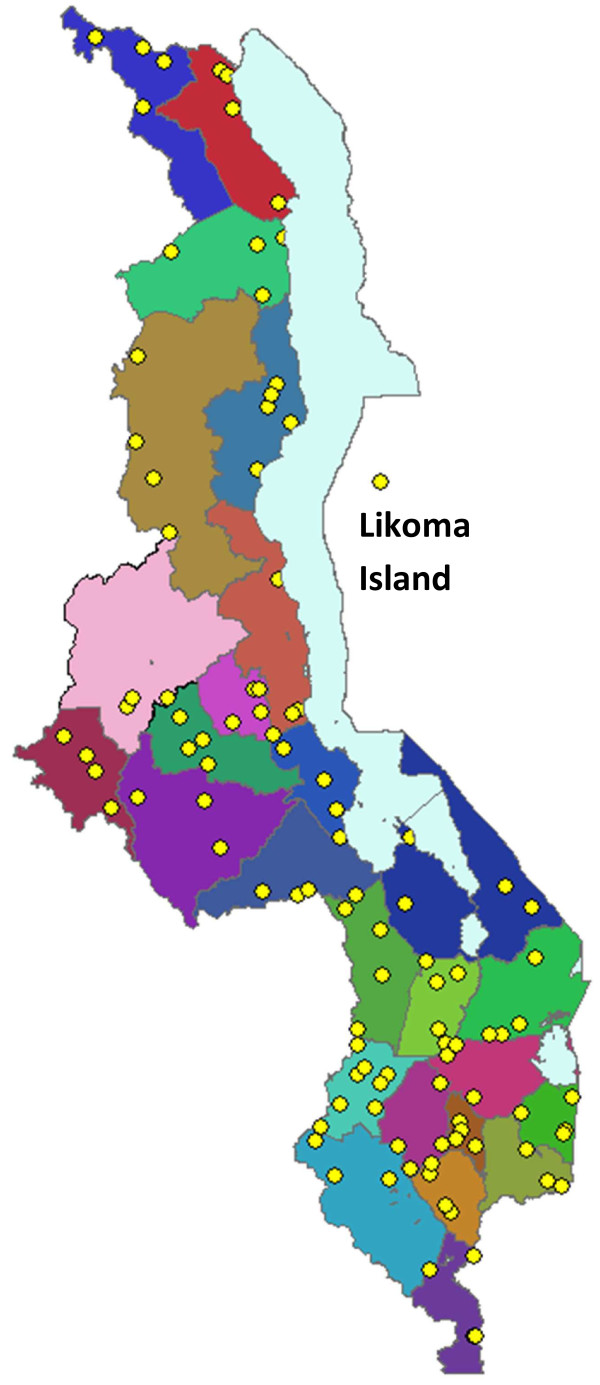
**Map of surveyed health facilities.** Note: Each yellow dot represents one surveyed facility. Districts are shaded in different colours.

Data were collected on 2,019 patients presenting for outpatient curative care and 136 health workers providing outpatient consultations on the survey day at the 107 facilities visited by survey teams (Table 1 and Figure 1). Most patients (86.5%) presented with illnesses involving fever (Table 2), which was more common among children aged < five years (94.1%) compared to patients aged ≥ five years (81.8%) (p < 0.0001). More than one-third of all patients (34.0%) had confirmed malaria. Confirmed malaria was significantly more common among patients aged < five years compared to those ≥ five years (45.5 *versus* 27.0%, p = 0.0004). Malaria was highest among those aged 5–14 years and significantly lower among patients aged 15 years and older (54.5% *versus* 17.2%, p < 0.0001). More than half of all patients (55.1%) reported that they spontaneously complained about fever to the clinician, with cough being the next most common complaint (40.0% of patients). Among patients not complaining of fever, clinicians asked about fever about one-third of the time (34.2%) and took patients’ temperatures 9.1% of the time; more than one-quarter of patients (27.7%) did not have their temperature or history of fever assessed by the clinician by any means (Table 2). Only 81.4% of health centres had a thermometer present and only 24.4% of facilities were able to perform malaria microscopy on the day of the team’s visit. Medical and clinical officers were significantly less likely to assess patients’ temperature or history of fever (51.0%) than medical assistants (73.8%), and nurses (82.6%) (p = 0.0005).

**Table 1:** Study sample

	**Facility ownership**		**Total**
	**Government**	**CHAM**	
Health facilities*	78	29	107
Health centre	**7**	1	8
District hospital	66	20	86
Rural hospital	5	8	13
**Health workers**	97	38	136
Medical/clinical officer	**7**	10	17
Medical assistant	82	19	101
Nurse	8	10	18
**Patients with complete interview and blood smear data**	1,645	374	2,019

*Non-surveyed include those not visited, (one in Likoma district) or those visited but not functional (one in Balaka district and another in Phalombe district).

**Table 2:** Patient demographics, clinical characteristics and clinician assessment and treatment, percentage

	**Patient age**			
	**<5 years %**	**5+ years %**	**p-value**	**Total %**
	***n=806***	***n=1209***		***n=2019***
**Demographics**				
Age in years, Mean (range)	1.9 (0.0, 5.0)	28.5 (5.0, 86.4)	–	**18.4 (0.0, 86.4)**
Male	51.4	37.8	<0.0001	**42.9**
**Clinical presentation**				
High temperature (≥37.5°C) during exit interview	38.7	21.2	<0.0001	**27.8**
Presented with an illness involving a fever*	94.1	81.8	<0.0001	**86.5**
Positive exit interview blood smear (BS)	45.8	28.7	0.001	**35.2**
Uncomplicated malaria prevalence (fever and positive BS)	45.5	27.0	0.001	**34.0**
**Spontaneously complained to health worker about:**				
Fever	79.7	40.1	<0.0001	**55.1**
Cough	56.2	30.0	<0.0001	**40.0**
Vomiting	20.8	8.3	<0.0001	**13.1**
Chills	5.6	13.9	<0.0001	**10.7**
Fatigue	5.7	1.0	<0.0001	**3.7**
**Health worker assessment of fever**				
Asked patient about fever^¶^	46.9	31.6	0.028	**34.2**
Took temperature^†^	19.5	7.0	0.001	**9.1**
Did not ask about fever or take temperature (and fever not reported by patient)	9.2	39.0	<0.0001	**27.7**
**Correct diagnosis and treatment of patients with confirmed malaria (according to exit interview BS)**	*n=269*	*n=358*		***n=629***
Health worker diagnosis of malaria	79.8	66.6	0.031	**73.2**
Correct treatment^¥^	72.7	61.5	0.066	**67.1**
**Overtreatment of patients without confirmed malaria**	*n=537*	*n=851*		***n=1,390***
Health worker diagnosis of malaria	44.0	31.5	0.069	**35.3**
ACT prescription^¥^	43.8	24.9	0.001	**30.9**

Note: data are weighted except for age ranges (unweighted).

*Includes positive responses for: 1) patient says illness involved a fever; 2) patient spontaneously mentioned fever complaint to health worker; 3) patient reported a symptom of fever to surveyor when probed, or temperature on re-examination was >=37.5°C.

^¶^If patient does not spontaneously report to health worker.

^†^If patient does not spontaneously report fever and health worker does not ask about fever.

^¥^ACT for most patients, or oral quinine for pregnant women in their first trimester or patients weighing less than 5 kg.

**Figure 2:**
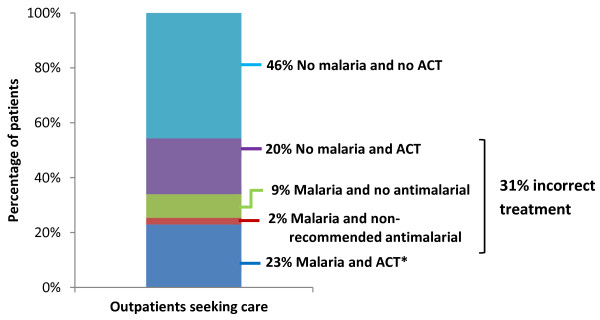
**Malaria and case management among outpatients attending publicly funded health facilities in Malawi (N = 2,019).** Note: Percentages are weighted. * Includes one pregnant patient in her first trimester who received oral quinine (correct treatment).

Among 629 patients with confirmed malaria, 73.2% were diagnosed with malaria by the clinician and 67.1% were prescribed correct malaria treatment. Among the 1,390 patients without confirmed malaria, 35.3% were diagnosed with malaria by the clinician and 30.9% were prescribed an ACT (or other appropriate anti-malarial), which is considered overtreatment (Table 2). Among all surveyed patients, 31% were incorrectly treated for malaria: 20% due to overtreatment and 11% due to non-receipt of first-line treatment among patients with malaria (Figure 2).

### Factors related to correct treatment of patients with confirmed malaria

Table 3 presents patient-level, health worker-level, health facility-level, and regional factors associated with correct malaria treatment. The strongest predictors of correct malaria treatment were patient-level symptoms. Adjusted for other factors, patients who spontaneously complained of fever to the health worker were 72% more likely to be correctly treated for confirmed malaria (p < 0.0001), and those who complained of chills were 30% more likely to get correct treatment (p = 0.008) (Table 3). However, those who complained of cough to the health worker were 27% less likely to be correctly treated for confirmed malaria (p = 0.001). Patients who had a high temperature (≥37.5°C) according to the exit interview measurement were 33% more likely to be correctly treated for confirmed malaria (p < 0.0001). Health worker type was significantly associated with correct malaria treatment. Medical assistants, a cadre with two years of medical training, were 54% more likely to correctly treat confirmed malaria compared to medical or clinical officers (p = 0.039). No other health worker factors, including previous training in either malaria case management or IMCI, were significantly related to correct treatment. Health worker diagnosis of malaria was not included in the model as it falls in the causal pathway of correct treatment, but was analysed as a separate outcome (see below). No health facility-level or regional factors were significantly related to correct malaria treatment among patients with confirmed malaria.

**Table 3:** Predictors of correct malaria treatment, among patients with confirmed malaria (N = 629)

		**Unadjusted prevalence ratio**	**Confidence interval**	**p-value**	**Adjusted prevalence ratio**	**Confidence interval**	**p-value**
Patient	**Male sex**	1.05	[0.93, 1.17]	0.531			
	**Spontaneous complaint to provider of:**						
	Fever	2.03	[1.44, 2.85]	<0.0001	1.72	[1.30, 2.29]	<0.0001
	Cough	0.74	[0.60, 0.92]	<0.0001	0.73	[0.61, 0.88]	0.001
	Vomiting	1.23	[1.08, 1.41]	<0.0001	1.03	[0.89, 1.20]	0.663
	Chills	1.32	[1.10, 1.60]	<0.0001	1.30	[1.07, 1.58]	0.008
	Fatigue	0.29	[0.07, 1.29]	0.104			
	**Age <5 years**	1.18	[0.98, 1.43]	0.085	1.08	[0.91, 1.28]	0.374
	**High temperature (≥37.5°C) according to exit interview**	1.48	[1.23, 1.78]	<0.0001	1.33	[1.19, 1.50]	<0.0001
Health provider	**Female health worker**	1.28	[1.04, 1.59]	0.022	1.23	[0.97, 1.55]	0.084
	**Type of clinician**						
	Medical officer/doctor/clinical officer	[Reference]	[Reference]	[Reference]	[Reference]	[Reference]	[Reference]
	Medical assistant	1.97	[1.26, 3.10]	0.004	1.54	[1.02, 2.31]	0.039
	Nurse	1.90	[1.16, 3.12]	0.012	1.18	[0.72, 1.92]	0.503
	**Copy of current malaria treatment guidelines**	0.82	[0.65, 1.03]	0.084	0.87	[0.73, 1.04]	0.130
	**Malaria-specific training**	1.00	[0.74, 1.35]	0.990			
	**Supervision in previous 6 months**	0.79	[0.63, 0.99]	0.042	1.10	[0.85, 1.43]	0.446
Health facility	**Type of facility**						
	District hospital	[Reference]	[Reference]	[Reference]			
	Health centre	1.27	[0.86, 1.87]	0.225			
	Community hospital	1.03	[0.53, 1.99]	0.938			
	**AL in stock for full day**	0.94	[0.71, 1.24]	0.660			
	**Thermometer present**	1.03	[0.75, 1.43]	0.841			
	**Functional microscopy**	0.93	[0.68, 1.25]	0.615			
	**CHAM-operated facility**	1.13	[0.92, 1.37]	0.235			
Region	**Region**						
	Northern	[Reference]	[Reference]	[Reference]			
	Central	0.98	[0.74, 1.31]	0.898			
	Southern	0.91	[0.70, 1.18]	0.472			

### Factors related to overtreatment of patients without malaria

Patients without confirmed malaria who complained to the health worker of fever, vomiting or chills were significantly more likely to be overtreated (p < 0.0001), while those who complained of fatigue were significantly less likely to be overtreated for malaria (p = 0.008) (Table 4). Compared to patients seen by medical or clinical officers, those without malaria seen by medical assistants or nurses were more than three times as likely to be overtreated, though these associations were only marginally significant (p = 0.063 and p = 0.053, respectively). No health facility-level or regional factors were significantly associated with overtreatment of patients without confirmed malaria.

**Table 4:** Predictors of malaria overtreatment (prescription of ACT to patients without confirmed malaria) (N = 1,390)

		**Unadjusted prevalence ratio**	**Confidence interval**	**p-value**	**Adjusted prevalence ratio**	**Confidence interval**	**p-value**
Patient	**Male sex**	1.27	[1.00, 1.60]	0.046	1.10	[0.92, 1.30]	0.276
	**Spontaneous complaint to provider of:**						
	Fever	2.64	[2.08, 3.33]	<0.0001	2.10	[1.71, 2.58]	<0.0001
	Cough	1.18	[0.90, 1.56]	0.233			
	Vomiting	1.74	[1.28, 2.38]	0.001	1.43	[1.20, 1.71]	<0.0001
	Chills	1.67	[1.32, 2.12]	<0.0001	1.66	[1.34, 2.04]	<0.0001
	Fatigue	0.35	[0.13, 0.93]	0.036	0.38	[0.19, 0.77]	0.008
	**Age <5 years**	1.82	[1.30, 2.57]	0.001	1.13	[0.91, 1.41]	0.258
	**High temperature (≥37.5°C) according to exit interview**	1.87	[1.52, 2.29]	<0.0001	1.37	[1.13, 1.66]	0.001
Health provider	**Female health worker**	1.32	[0.86, 2.03]	0.20			
	**Type of clinician**						
	Medical officer/doctor/clinical officer	[Reference]	[Reference]	[Reference]			
	Medical assistant	3.75	[0.93, 15.16]	0.063	3.63	[0.93, 14.17]	0.063
	Nurse	4.39	[1.01, 19.06]	0.048	3.99	[0.98, 16.24]	0.053
	**Copy of current malaria treatment guidelines**	0.86	[0.60, 1.24]	0.424			
	**Malaria-specific training**	1.20	[0.70, 2.06]	0.514			
	**Supervision in previous 6 months**	0.89	[0.53, 1.48]	0.653			
Health facility	**Type of facility**						
	District hospital	[Reference]	[Reference]	[Reference]			
	Health centre	3.21	[0.95, 10.92]	0.061			
	Community hospital	2.16	[0.60, 7.78]	0.234			
	**AL in stock for full day**	0.87	[0.55, 1.40]	0.572			
	**Thermometer present**	1.02	[0.60, 1.73]	0.940			
	**Functional microscopy**	0.76	[0.44, 1.30]	0.311			
	**CHAM-operated facility**	1.31	[0.83, 2.07]	0.242			
Region	**Region**						
	Northern	[Reference]	[Reference]	[Reference]			
	Central	1.26	[0.63, 2.52]	0.515			
	Southern	1.28	[0.78, 2.10]	0.328			

### Factors related to health worker diagnosis of malaria

The most important determinants of whether patients received a malaria diagnosis from health workers were the clinical symptoms reported by patients to health workers (Table 5). Among all patients, those who spontaneously complained about fever were 86% more likely to be diagnosed with malaria (p < 0.0001), as were those complaining about vomiting or chills (22 and 35% more likely to be diagnosed with malaria, p = 0.001 and p < 0.0001, respectively). Patients presenting with a complaint of cough, however, were 19% less likely to be diagnosed with malaria (p = 0.001). The 6% of patients complaining about fatigue were also 51% less likely to be diagnosed with malaria by health workers (p = 0.045). Adjusted for other factors, lower-level cadres of health workers were about twice as likely as medical or clinical officers to diagnose patients with malaria. Patients attending facilities where AL was in stock for the full day were 15% less likely to be diagnosed with malaria (p = 0.014), and those attending CHAM facilities were 25% more likely to be diagnosed with malaria (p = 0.001).

**Table 5:** Predictors of malaria diagnosis by clinician (N = 2,019)

		**Unadjusted prevalence ratio**	**Confidence interval**	**p-value**	**Adjusted prevalence ratio**	**Confidence interval**	**p-value**
Patient	**Male sex**	1.25	[1.03, 1.52]	0.025	1.13	[0.99, 1.29]	0.067
	**Spontaneous complaint to provider of:**						
	Fever	2.34	[1.91, 2.86]	<0.0001	1.86	[1.59, 2.18]	<0.0001
	Cough	0.86	[0.77, 0.97]	0.011	0.81	[0.73, 0.91]	0.001
	Vomiting	1.58	[1.32, 1.89]	<0.0001	1.22	[1.09, 1.38]	0.001
	Chills	1.47	[1.26, 1.71]	<0.0001	1.35	[1.18, 1.54]	<0.0001
	Fatigue	0.37	[0.13, 1.03]	0.058	0.49	[0.24, 0.98]	0.045
	**Age <5 years**	1.49	[1.19, 1.86]	0.001	1.04	[0.90, 1.20]	0.602
	**High temperature (≥37.5°C) according to exit interview**	1.84	[1.59, 2.14]	<0.0001	1.41	[1.23, 1.60]	<0.0001
Health provider	**Female health worker**	1.02	[0.74, 1.40]	0.909			
	**Type of clinician**						
	Medical officer/doctor /clinical officer	[Reference]	[Reference]	[Reference]	[Reference]	[Reference]	[Reference]
	Medical assistant	2.57	[1.56, 4.25]	<0.0001	2.14	[1.40, 3.27]	0.001
	Nurse	2.64	[1.53, 4.54]	0.001	1.8	[1.16, 2.79]	0.009
	**Copy of 2007 malaria treatment guidelines**	1.11	[0.87, 1.40]	0.401			
	**Malaria-specific training**	1.03	[0.74, 1.44]	0.862			
	**Supervision in previous 6 months**	1.14	[0.88, 1.46]	0.312			
Health facility	**Type of facility**						
	District hospital	[Reference]	[Reference]	[Reference]			
	Health centre	1.92	[0.97, 3.79]	0.060			
	Community hospital	1.89	[0.92, 3.88]	0.082			
	**AL in stock for full day**	0.72	[0.57, 0.90]	0.005	0.85	[0.75, 0.97]	0.014
	**Thermometer present**	0.82	[0.62, 1.07]	0.135			
	**Functional microscopy**	0.69	[0.50, 0.95]	0.025	0.91	[0.71, 1.16]	0.426
	**CHAM-operated facility**	1.37	[1.06, 1.76]	0.015	1.25	[1.09, 1.43]	0.001
Region	**Region**						
	Northern	[Reference]	[Reference]	[Reference]			
	Central	1.17	[0.78, 1.78]	0.443			
	Southern	1.15	[0.86, 1.54]	0.345			

To further explore the determinants of correct malaria treatment, the sensitivity and specificity of health worker diagnosis of malaria, and correct treatment among patients they diagnosed with malaria were examined. The sensitivity of health worker diagnosis of malaria was only 73.2% and the specificity 64.7% compared to the gold standard derived from the reference blood smear. Given the prevalence of malaria, the positive predictive value of a health worker diagnosis of malaria was only 52.0%. Among patients diagnosed by health workers as having malaria, the sensitivity of correct treatment, i e, the proportion prescribed an appropriate anti-malarial, was 83.3% and the specificity was 94.2%, meaning that only 6% of all patients received ACT (or other appropriate malaria treatment) if they were not diagnosed with malaria by the health worker. Among the 158 patients diagnosed with malaria by health workers but not receiving correct treatment, 37.8% received no malaria treatment at all and 62.2% were prescribed a non-recommended treatment, including SP, injectable quinine and oral quinine (Additional file 1). Of the 59 patients diagnosed with malaria but receiving no antimalarial, 10 had a negative diagnostic test, and it is unclear why the remaining 48 patients received no antimalarial prescription. A separate analysis of factors related to correct treatment of patients diagnosed by health workers with malaria found that other than fever, which increased the probability of correct treatment, no factors at the patient-, health worker-, or health facility levels were significantly associated with correct treatment (Additional file 2).

## Discussion

Only two-thirds of patients with confirmed malaria were correctly treated, and nearly one-third of patients without confirmed malaria received malaria treatment, resulting in 31% of all outpatients being incorrectly treated for malaria. The most important predictors of correct malaria case management, as well as overtreatment of patients without malaria, were patients’ presenting clinical signs and symptoms. Spontaneous complaints of fever or chills, as well as high measured temperature were significantly related to correct treatment as well as overtreatment of malaria. However, spontaneous mention of cough was associated with incorrect treatment for patients with confirmed malaria.

In a setting without universal malaria diagnostics, such as some of the facilities included in this study, health workers must primarily rely on clinical signs and symptoms to diagnose and treat patients. However, assessment of fever was not consistent, and health workers did not ascertain fever or history of fever in more than one-quarter of patients. RDTs hold potential to improve case management, but only if health workers improve the first critical step of identifying febrile patients who should be tested.

Even with systematic elicitation of symptoms, purely clinical diagnostic algorithms for malaria generally do not perform well [10,24]. In addition to general non-specificity and overlap with multiple other potential causes, the symptoms of malaria have significant overlap with pneumonia symptoms in children [25,26] and other febrile illnesses in all ages. Previous studies have shown higher rates of pneumonia symptoms (cough or difficult breathing plus raised respiratory rate) in RDT-negative *versus* RDT-positive children presenting to a health clinic in Tanzania; however, these symptoms were common in both groups of children: 51.6 *versus* 30.4%, p < 0.0001 [27]. This might imply that discrimination of malaria diagnosis based on history of cough is somewhat rational, though still not justified given the high prevalence of cough among febrile patients, and association of cough with incorrect malaria treatment in this study. To the authors’ knowledge, this is the first study demonstrating significantly worse malaria treatment for patients presenting with a complaint of cough, although one study from Kenya found a negative association between patient complaint of cough and diagnostic testing for malaria [28]. Health workers should be reminded about the potential for co-morbidities and to perform diagnostic testing of all febrile patients, including those with cough.

Besides clinical presentation, the only factor significantly associated with malaria case management quality was health worker type, with medical assistants and nurses, who provide the bulk of outpatient care in Malawi, more likely to prescribe first-line anti-malarial treatment to patients compared to clinical or medical officers. Other studies have found that lower-level cadres of health workers are more likely to adhere to guidelines, including for malaria treatment [20], than more qualified ones, who may rely more on their clinical experience and intuition. In this analysis, no other health worker-level, facility-level, or regional factor, including training, supervision, equipment, drug stocks, or availability of treatment guideline, was related to correct malaria treatment or overtreatment. This does not imply that factors such as supervision and training are not important for malaria case management, but this study was not necessarily designed to measure the quality of these factors in a nuanced way.

With low availability of diagnostic testing in this setting, health worker malaria diagnosis is a critical step in the pathway to correct treatment. However, correct treatment among patients with health worker-diagnosed malaria was still only 83%, with slightly more than two-thirds of those not correctly treated receiving no malaria treatment at all and the remainder being prescribed a non-recommended anti-malarial. Interestingly, no factors analysed were significantly predictive of correct treatment among patients diagnosed by health workers with malaria, underscoring the importance of reinforcing prescription of appropriate first-line treatment for all patients diagnosed with malaria.

With the reliance on clinical diagnoses at the time of this survey, RDTs could potentially improve malaria case management in Malawi. Experience in introducing RDTs has shown that they can decrease consumption of ACT [29-31], but some studies found that providers did not test patients systematically [22] or did not treat according to RDT results [32,33].

RDT provision in settings without high testing rates, or provision of insufficient quantities of RDTs, can have unintended consequences of decreasing correct treatment of patients with malaria, as presumptive treatment may also decline [22]. Initial and refresher training on RDTs provides an opportunity to reinforce assessment and testing algorithms. Additional attention should be paid to health worker adherence to RDT results, which has been problematic in this and other settings [34,35]. Attention must also be given to prioritizing guidelines and training for patients who test negative for malaria, which is a global challenge [8,27,36] that will increase as malaria prevalence declines. Ensuring that anti-malarials are not prescribed for negative RDT results should help reduce the substantial overtreatment of patients without malaria (31% in this study) and help appropriately treat non-malarial causes of fever.

Interventions to improve health worker case management behaviours include training/refresher training, enhanced supportive supervision, job aids, audit and feedback sessions, and other strategies, although definitive evidence on the most effective strategies to improve health worker performance is lacking [37]. Many countries have implemented training to improve malaria case management, although results have been mixed, with some studies showing positive improvements [38,39] and others indicating little to no effect of training [40,41]. Recent evidence suggests that newer strategies such as text message reminders to health workers on key case management practices might substantially improve provider behaviors at relatively low costs [42,43]. Community education efforts to emphasize the importance of pro-actively mentioning fever to health workers when seeking care should also be considered, given the importance of patient complaints in directing diagnosis.

This study had several limitations. First, the study was conducted during the high-transmission season and might not represent case management patterns during other times of the year. Second, only publicly funded health facilities, including CHAM facilities, were surveyed, limiting generalizability to these facilities. However, these facilities provide the vast majority of care in Malawi, with the for-profit private sector providing an estimated 3% of health services [21]. Third, study results might have been subject to bias from the “Hawthorne” effect, whereby health workers perform better when they are aware their actions are being studied [44]. However, the findings nonetheless indicated significant gaps in case management.

This study found that health worker case management decisions were primarily driven by patient-level complaints and health workers are not systematically identifying fever or history of fever in patients. In addition to training on the new diagnostic guidelines along with the RDT rollout, policymakers and malaria programme managers should consider various forms of job aids for easy reference, enhanced supportive supervision to reinforce systematic testing of patients and adherence to test results, as well as more novel approaches such as text message reminders to improve malaria case management. Efforts to rigorously evaluate these approaches will help ensure that these investments translate into better malaria diagnosis and treatment in Malawi.

## Competing interests

The authors declare that they have no financial or non-financial competing interests.

## Authors’ contributions

LCS oversaw data collection, conducted data analysis and produced the first draft of the manuscript. JC oversaw data collection and conducted data analysis. AW assisted with study design and designed data collection instruments. ML (MAC) oversaw data collection and ML (NMCP) conducted data analysis. JS (NMCP), DA and DM provided important input into the study design and manuscript draft. JS conceived of the study idea and analysis plan. All authors have seen and approved the final draft of the manuscript.

## Supplementary Material

Additional file 1 Cascade of health worker diagnosis and treatment of patients presenting for case, by true malaria status. Click here for file

Additional file 2 Predictors of incorrect treatment among patients diagnosed with malaria by clinicians (N=999). Click here for file
