# Supplementary material for: Patient-, health worker-, and health facility-level determinants of correct malaria case management at publicly funded health facilities in Malawi: results from a nationally representative health facility survey
Source: Malar J. 2014 Feb 20;13:64. doi: 10.1186/1475-2875-13-64 (PMC3938135; doi:10.1186/1475-2875-13-64)
Supplement: Additional file 1 — Cascade of health worker diagnosis and treatment of patients presenting for case, by true malaria status. [file 1475-2875-13-64-S1.docx]

* Gold-standard malaria defined as fever/history of fever plus parasitemia on exit interview blood smear.

Note: N’s not weighted; percentages weighted. ACT refers to ACT (for most patients) or oral quinine for pregnant women or patients weighing < 5 kg. Other AM refers to other antimalarial and includes oral quinine, injectable quinine, and SP.

**All patients**

N=2,019

**No HW malaria**

**diagnosis** n=1,008

**52%**

**HW malaria diagnosis**

n=1,011

**48%**

**Gold standard malaria***

n=482

**52%**

**No gold standard malaria***

n=529

**48%**

**ACT**

n=425

**87%**

**No ACT**

n=57

**13%**

**ACT**

n=428

**79%**

**No ACT**

n=101

**21%**

**ACT**

n=11

**13%**

**No ACT**

n=136

**87%**

**ACT**

n=25

**4%**

**No ACT**

n=836

**96%**

**Gold standard malaria***

n=147

**18%**

**No gold standard malaria***

n=861

**82%**

**Other AM**

n=39

**67%%**

**Other AM**

n=58

**59%**

**No AM**

n=42

**41%**

**No AM**

n=17

**33%**

**Other AM**

n=5

**3%%**

**Other AM**

n=10

**1%**

**No AM**

n=797

**99%**

**No AM**

n=130

**97%**
