# Supplementary material for: Patient-, health worker-, and health facility-level determinants of correct malaria case management at publicly funded health facilities in Malawi: results from a nationally representative health facility survey
Source: Malar J. 2014 Feb 20;13:64. doi: 10.1186/1475-2875-13-64 (PMC3938135; doi:10.1186/1475-2875-13-64)
Supplement: Additional file 2 — Predictors of incorrect treatment among patients diagnosed with malaria by clinicians (N=999). [file 1475-2875-13-64-S2.docx]

**Appendix 2:** Predictors of incorrect treatment among patients diagnosed with malaria by clinician (N 999)

|  |  | Unadjusted prevalence ratio | Confidence interval |
| --- | --- | --- | --- |
| Patient | Male sex | 0.98 | [0.68, 1.42] |
|  | Spontaneous complaint to provider of: |  |  |
|  | Fever | 0.43 | [0.25, 0.74] |
|  | Cough | 1.03 | [0.69, 1.53] |
|  | Vomiting | 1.01 | [0.76, 1.35] |
|  | Chills | 0.70 | [0.36, 1.34] |
|  | Fatigue | 1.96 | [0.86, 4.44] |
|  | Age <5 years | 0.53 | [0.28, 1.02] |
|  | High temperature (≥37.5°C) according to exit interview | 0.67 | [0.37, 1.22] |
| Health provider | Female health worker | 0.59 | [0.26, 1.35] |
|  | Type of clinician |  |  |
|  | Medical officer/doctor /clinical officer | [Reference] | [Reference] |
|  | Medical assistant | 0.58 | [0.25, 1.34] |
|  | Nurse | 0.41 | [0.15, 1.09] |
|  | Copy of 2007 malaria treatment guidelines | 1.69 | [0.76, 3.76] |
|  | Malaria-specific training | 0.85 | [0.28, 2.54] |
|  | Supervision in previous 6 months | 1.12 | [0.48, 2.60] |
| Health facility | Type of facility |  |  |
|  | District hospital | [Reference] | [Reference] |
|  | Health centre | 0.62 | [0.36, 1.05] |
|  | Community hospital | 1.26 | [0.30, 5.29] |
|  | AL in stock for full day | 0.60 | [0.23, 0.61] |
|  | Thermometer present | 0.66 | [0.23, 1.87] |
|  | Functional microscopy | 1.45 | [0.59, 3.58] |
|  | CHAM-operated facility | 0.87 | [0.42, 1.79] |
| Region | Region |  |  |
|  | Northern | [Reference] | [Reference] |
|  | Central | 1.29 | [0.46, 3.59] |
|  | Southern | 1.41 | [0.61, 3.29] |
